# CTO Pathophysiology: How Does this Affect Management?

**DOI:** 10.2174/1573403X10666140331142349

**Published:** 2014-05

**Authors:** John Irving

**Affiliations:** Ninewells Hospital, Dundee UK

**Keywords:** Angioplasty, chronic total occlusion, collateral, pathophysiology.

## Abstract

Chronic total occlusion (CTO) pathophysiology has been described in a few, small studies using post mortem histology, and more recently, *in vivo* intravascular ultrasound (IVUS) to analyse the constituents of occluded segments. Recent improvements in equipment and techniques have revealed new insights into physical characteristics of occluded coronaries, which in turn enable predictable procedural success. The purpose of this review is to consider the published evidence describing CTO pathophysiology from the perspective of the hybrid algorithm approach to CTO PCI.

*Methods*: Literature searches using “Chronic Occlusion”, “angioplasty”, and” pathology” as keywords. Further searches on “coronary” “collateral”, “Viability”. Bibliographies were scrutinised for further key publications in an iterative process. Papers describing animal models were excluded.

## INTRODUCTION

Many patients with coronary artery disease have chronically occluded vessels. Typical prevalence rates from retrospective surveys of coronary angiography series were between 33% and 52% of patients with ischaemic heart disease (IHD) [1, 2]. A recent, comprehensive survey from 3 large Canadian Institutions found chronic total occlusions (CTO) in 18% of patients undergoing non emergent angiography [3]. In these studies, a minority (40%) of patients had a prior history of myocardial infarction, and fewer than one third had Q waves in the distribution of the occluded vessel. Furthermore left ventricular (LV) function was normal in the majority of CTO patients.

The phenomenon of occluded vessels upstream of undamaged myocardium depends upon an adequate alternative blood supply. Coronary collaterals delivering blood from unobstructed coronaries to the distal bed of CTO vessels have been well characterised for many years [4]. Indeed, they may have been described in the 17^th^ century by the English physiologist Richard Lower as a result of direct experiments [5]. This circulation is not present in health, but develops as a result of pressure mediated dilatation of arterioles. This process can be remarkably effective, often preventing any detectable infarction in the occluded vessel territory. However this process requires approximately 2 weeks to reach maturity and acute thrombotic occlusion often occurs more rapidly than the process of arteriogenesis required to develop a completely protective collateral circulation.

PCI is carried out to a minority of chronic occlusions; many patients can be managed to their satisfaction with medical therapy alone. However, the perception among general cardiologists that CTO PCI is difficult, expensive or unnecessary still carries disproportionate weight, despite recent technical developments and increased success rates. The essential difficulty in CTO PCI is achieving a guidewire connecting distal and proximal lumens, because the occluded segment is resistant to penetration.

This review outlines the evidence that underpins our understanding of the pathophysiology of chronically occluded coronary arteries. This leads to a summary of the generally accepted narrative of CTO formation and consolidation. Factors that determine the final morphology of the lesion are not well understood, and unanswered issues will be outlined. A short summary of collateral biology follows. The review concludes by discussing the facets of pathophysiology that influence tactical decisions within the hybrid algorithm, and some intriguing alternative techniques. A detailed understanding of current knowledge of the foundations of chronic coronary occlusion biology may help future developments in the rapidly developing field of CTO PCI.

## POST MORTEM PAPERS

There are two important case series published, and each will be described in detail. It is likely that post mortem studies have been used during development of CTO specific devices, but these remain the commercial property of the companies owning device patents, and have not been published in peer reviewed journals. 

The first post mortem study specific to human coronary CTO was published in 1993 [6]. Katsuragawa M, *et al.* described post mortem findings in ten cases of CTO. Age of occlusion, estimated from the clinical history of myocardial infarction in the territory of the occluded coronary artery, was more than 1 year prior to death in each case. Each CTO had been demonstrated by coronary angiography less than three months before death. Histological examination of the occluded segment showed fibrous tissue, atheroma with or without calcification, vascular tissue and infiltrates of lympohocytes. The key finding was very different tissue composition in vessels where the proximal end of occlusion appeared to be tapered on angiography, compared to vessels where the proximal stump appeared blunt. Occlusions with a tapered cap were shorter, and less likely to have sidebranches at the proximal cap (1/5 versus 4/5 non- tapered occlusions). The tissue content of tapered occlusions was characteristically looser fibrous tissue, with prominent neovascularisation and recanalization. This dichotomy of CTO composition did not appear to relate to age of occlusion or length of occluded segment in this study, although the numbers were small.

Srivatsa SS, *et al.* [7] published the seminal paper, describing histological findings in 96 occluded vessels in 61 patients who underwent post mortem examination within three months of pre-mortem coronary angiography. 

Age of the CTO was determined in patients who had had serial angiograms by time from progression from a subtotal lesion to occlusion, or patients with clear documentation of myocardial infarction preceding documentation of CTO in the infarcted territory. Coronary arteries were sectioned and samples from proximal, mid and distal parts of the occluded segment were examined in detail. Specific components were identified by specific stains, and quantified according to location: lumen, intimal plaque, media and adventitia. 

Older CTOs had fewer foam cells and macrophages and greater calcification and fibrosis. Neovascularisation of plaque was associated with inflammatory cells. Recanalisation was extensive and found in 59% of CTOs without relating to CTO age in this series. Plaque neovasculature was also observed in most CTOs (85% of those older than one year), but connections between plaque vessels and recanalised lumen were rarely demonstrable.

Interestingly, variation in plaque composition associated with lesion age was independent of the histologic degree of lumen stenosis found in this study; each broad plaque type, soft, mixed, or hard were equally represented. All vessels were found to be >90% occluded at autopsy, or were rejected from this analysis. Strikingly 49% of vessels were less than 99% occluded by histological criteria, despite all having total angiographic occlusion. These functional occlusions were more prevalent in younger CTOs.

In summary, there are two important factors that relate to feasibility of crossing the occlusion with a wire:

- Plaque composition, which has a straightforward relationship with lesion age, with gradual replacement of cholesterol and foam cells with fibrous and calcific material.
- The extent of vessel recanalization, which is associated with looser fibrous tissue, and less resistance to wire passage, does not seem to have a clear relationship with age, nor lesion length.

## I*N VIVO* INVESTIGATION BY IVUS

Fuiji *et al*. published an excellent paper [8] describing IVUS findings in 83 CTOs interrogated with IVUS immediately after antegrade wire crossing, and small calibre balloon inflation. Procedures were performed in 4 Japanese centres with procedures performed between 2003 and 2005. 

This study found nearly all lesions contained calcium (96%) although in many, (68%), this was mild. The authors were able to define the proximal cap according to angiographic landmarks, and observing abrupt morphology change on IVUS. The proximal cap was a location where calcium was concentrated, particularly in blunt stump CTOs. A calcified arc was demonstrated in the wall opposite the sidebranch in 74% of this morphology. A smaller proportion had calcification found perpendicular to the sidebranch origin, and a small number were found on the ipsilateral aspect as the side branch. 

## IVUS CHANGES WITH TIME 

Suzuki *et al*. [9] described IVUS findings in 79 CTO lesions and found moderately strong correlations between lesion age and indices of calcification assessed by IVUS. Some very recent CTOs were heavily calcified suggesting that the CTO had arisen in a vessel with well entrenched atheroma.

## IVUS VH

New insights into tissue characteristics in CTO have been recently published by Guo *et al*., [10] analysing virtual histology IVUS images of a series of 50 CTOs treated by a single operator. Spectral analysis of ultrasound signals is used to classify plaque components as fibrotic, fibro-fatty calcified and necrotic core [11]. Areas of necrotic core were equally prevalent in RCA lesions and LAD lesions, but maximum necrotic core was slightly higher in longer than shorter lesions (41% vs 33%). There was no difference in tissue characteristics proximal, middle and distal CTO segments.

The authors defined two distinct CTO VH phenotypes. VH-fibroatheroma was the more prevalent pattern seen in 84% of lesions, and defined according to presence of confluent necrotic core in contact with the dilated guidewire track. The majority of these lesions (83%) had VH fibroatheroma occupying the length of the occluded segment, and small numbers had patches of this VH pattern. Lesions lacking these features had less necrotic core (19% vs 28%), less dense calcium (5% vs 12%) more fibrofatty (15% vs 2%) and fibrous tissue, at the site of maximum necrotic core, and very similar findings at the maximum plaque burden site.

Age of CTO was calculated but not disclosed in the paper; they note that a greater prevalence of fibrocalcific material was reported in the Srivasta study [7], which sampled older CTOs; 29% at least one year and 35% at least 5 years in duration.

VH fibroatheroma was characteristic of acute coronary syndrome lesions in non CTO lesions [12]; the authors suggest the majority of CTOs result from ACS lesions, and a minority from progression of atheroma. The reliability of the estimate of proportion of lesion type will be influenced by the case mix of the operator. This case series was performed in 16 separate institutions by a world expert. Cases selected to demonstrate particular techniques might plausibly over or underestimate relative prevalence of lesion type. 

## FEATURES OF CORONARY OCCLUSION IDENTIFIED BY PROCEDURE BEHAVIOUR

The essential difficulty in CTO PCI is achieving a guidewire traversing the occluded segment from proximal lumen to distal lumen. A secondary difficulty is advancing equipment along the wire path. Comprehensive accounts of techniques to overcome these issues are published in other articles in this series.

## PROXIMAL CAP

Frequently, CTO segments have a highly resistant proximal end, at the point of transition from patent vessel to occlusion. This has been identified on IVUS studies, and is often calcified. Interestingly VH characteristics did not seem to vary along the occluded segment in the single published study [10], and more studies would be of interest.

## DISTAL CAP

An analogous distal cap is recognised as a point of difficulty and resistance during wire passage. Considered to be less resistant than the proximal cap [13, 14], and conceptually important for the development of retrograde techniques; the distal cap is not described in the key papers summarised above.

## SUBINTIMAL SPACE

Longitudinal passage of an angioplasty guidewire in the media or adventitia is possible in any coronary artery. This is rarely helpful in a patent vessel, but enables rapid progress through a low resistance (potential) space, bypassing highly resistant tissue in the occluded lumen. Reliable vessel tracking dissection is achieved by looping a wire and pushing with the loop. This “knuckle wire” remains in the subintimal space, and though it may dissect a plane into a sidebranch, will perforate the vessel in exceptional circumstances only. The subintimal space is constrained by fibrous and calcified tissue derived from atheroma on the luminal aspect, and elastic adventitia on the external aspect of the vessel. Post mortem images of haematoma in a subintimal wire track have been published [15]. 

One limitation of this approach is reduction and loss of opacification of the distal vessel, which is likely to be due to haematoma in the subintimal space compromising filling. Bleeding might be from disruption of neovascularisation channels, or filling of the space with blood at systemic arterial pressure. The recently described STRAW technique [16], where occlusion of the entry segment and aspiration though an inflated OTW balloon can lead to restoration of distal visualisation suggests that filling of the space under arterial pressure is the most important factor.

## INTRALUMINAL TRACKING OF MICROCHANNELS

Neovascularisation channels are approximately 200 microns in diameter, which is slightly smaller than the tip of a tapered guidewire (for example fielder XT, Asahi Intecc, Japan). Every CTO operator has experience of progress with gentle probing with a soft tipped wire, leading to rapid wire passage and a short case. Strauss *et al*. suggested [17] that these cases have developed a potential guidewire track through longitudinal reconstruction of neovascularisation, or that they are illusory CTOs. 

The micro-vessel tracking hypothesis has been supported by a technique described in a short series by Carlino and co-authors [18]. They used a stiff guidewire to penetrate the proximal cap, and advanced an OTW balloon into the body of the occlusion. The central lumen was used to deliver GTN and contrast. Thirty-two lesions were attempted, in 19 visualisation of the distal vessel was achieved via the OTW catheter and the lesion was wired with a floppy wire, in 12 a dissection occurred, although the lesion was successfully treated in seven of these. One patient developed a peri-procedural myocardial infarction. 

Professor Strauss has further developed this hypothesis by demonstrating the potential of collagenase as a therapeutic agent in CTO PCI. The expectation is that enzymatic degredation of connective tissue will enable rapid and reliable wire crossing. A dose finding study reported promising clinical results [19]. Twenty patients with at least one prior failure to treat by conventional techniques were treated. Initially an OTW balloon was advanced to the proximal cap, inflated, and collagenase was administered via the guidewire lumen (8 patients). In the subsequent 12 patients a microcatheter was advanced into the lesion, and treatment was administered directly into the occluded segment. Patients returned to the lab the following day and lesion crossing was successful in 75% with no adverse events reported. A larger study is pending. http://clinicaltrials.gov/ct2/show/ NCT01753180

## IVUS INVESTIGATION OF WIRING PATH

The large series of Japanese cases reported by Fujii *et al.* found 34% of cases had intramural haematoma [8], implying guidewire probing of media, but in all cases post stent implantation the images demonstrated that the wire and IVUS catheter were in the true lumen throughout the treated segment. 

Different findings were reported in an analysis of cases from a single, US operator. (Dr Lombardi). This paper [20] reviewed IVUS images from 26 consecutive CTO patients treated in 2007 acquired after wiring and 1.5mm predilatation, or 1.25mm burr rotablation in 2 patients. Subintimal wire tracking was demonstrated in 45% of cases, with a greater tendency to occur in longer lesions, and more difficult procedures in which screening time, procedure time and contrast doses were all increased. In these cases where subintimal tracking occurred, this was found to extend to the adventitia in a small majority of patients (58% versus 42% confined to subintima). Interestingly subintimal tracking was also a feature of re-attempts, implying that the initial operator had stopped once establishing a sub-intimal position, and the second wire would tend to follow the initial track, but with persistence, the distal lumen could be achieved. 

## ACCEPTED NARRATIVE OF CTO FORMATION, AND DEBATABLE ISSUES

**Fig. (1):**
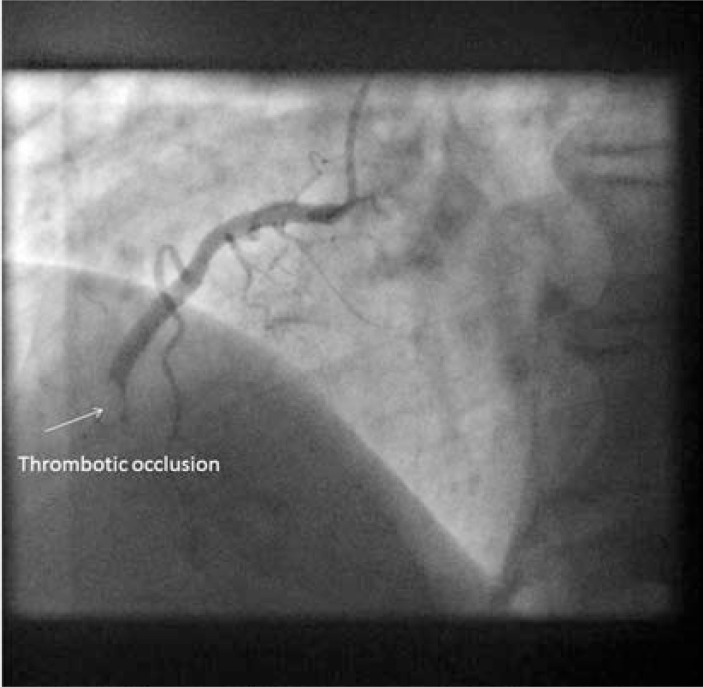
Image of coronary angiogram of RCA from late presenting STEMI. Normal LV function. Bulky thrombotic occlusion extending to small calibre marginal branch.

**Fig. (2):**
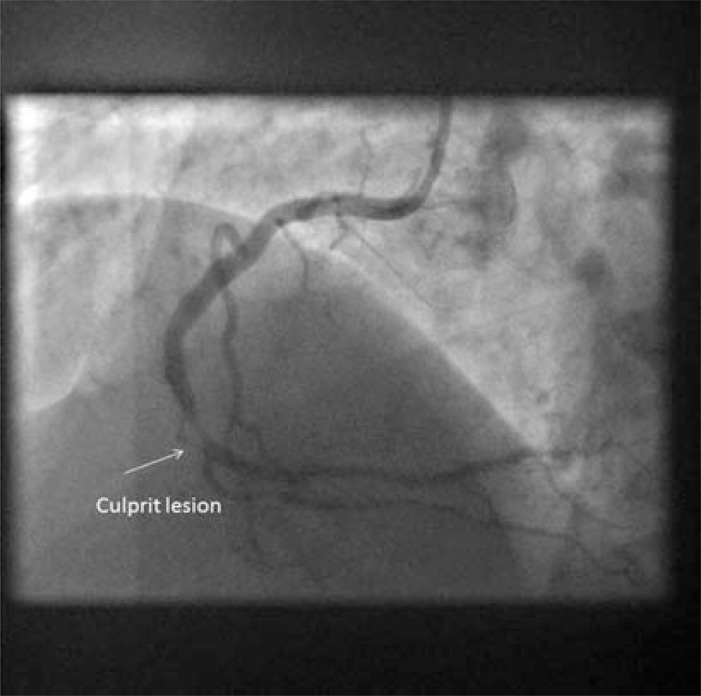
Coronary angiogram following aspiration thrombectomy. Culprit lesion 15mm distal to proximal margin of occlusive thrombus.

An expert conference on CTO angioplasty in 2004 developed a state of the art review, including a detailed narrative of CTO development [21]. This account developed an overview of the biology of acute coronary occlusion, from the human papers, existing animal models, and the experience of the assembled faculty. In essence, the occluded vessel results from plaque rupture followed by thombotic coronary occlusion and organisation of thrombotic material into the CTO. Frequently, thrombus will be propagated retrograde from the point of occlusion to a major sidebranch (Figs. **1**and **2**). The occluded segment remains biologically active with recanalization, neovascularisation, and inflammation within the occlusion resulting in different composition of CTOs according to age. The proximal limit of thrombus propagation is the site of proximal cap development, with fibrous and calcific atheroma forming at the site of high sheer stress, as flow channels down the side-branch. No rival account has been published, but questions remain regarding the details of the processes determining CTO morphology.

## DO CTOS DEVELOP FROM ACUTE OCCLUSION ON MINOR PLAQUES?

Guo *et al.* (10) have suggested that the predominant VH characteristic of CTO segments containing confluent necrotic core, and that this is analogous to findings in non CTO vessels. Variable correlation between VH imaging and histology has been reported [22-24] and has, for practical and ethical reasons, not been verified in human CTO lesions, and the impact of artefact from predilatation is unknown. Imaging at a single time point may not be representative of the morphology determining processes. If rupture of vulnerable plaque is the catalyst for CTO creation, one can infer that occlusive ACS lesions frequently develop over a time course that can enable collateral development and protection from infarction. 

## WHAT DETERMINES LENGTH OF OCCLUDED SEGMENT? 

There is no consensus on what determines location of proximal or distal cap relative to the initial point of occlusion. Does thrombus propagate up and downstream from the point of occlusion? Is there regression of thrombus? Why do some caps cleave to major sidebranches and others seem to stop mid vessel? The distal cap is well recognised clinically, but not well described in pathological or IVUS studies.

## IS LESION LENGTH ACCURATELY DETERMINED BY ANGIOGRAPHY?

Simultaneous contalateral injections with a second guide catheter often demonstrate a shorter occlusion than diagnostic angiography performed with a single catheter. Collateral filling is often variable between different runs. Furthermore, it may be that absence of contrast flow in the occluded segment is due to a highly stenosed segment, but apparent occlusion is much longer through lack of collateral supplied opacification of a diseased but non occlusive segment. 

## WHAT DETERMINES A TAPERED PROXIMAL CAP?

**Fig. (3):**
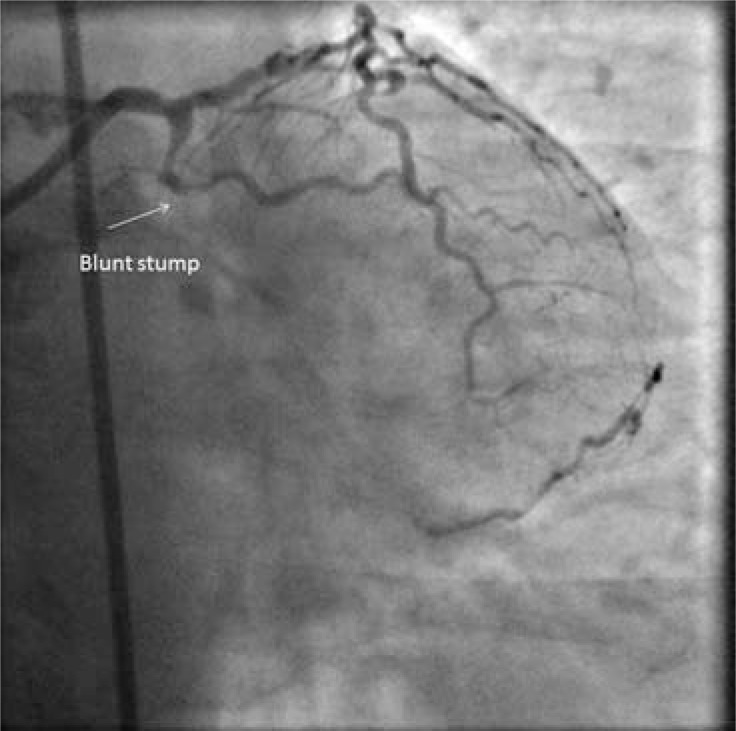
Still from angiogram of CTO of circumflex. Epicardial collaterals from diagonal. Symptoms predated this investigation by 6 months.

**Fig. (4):**
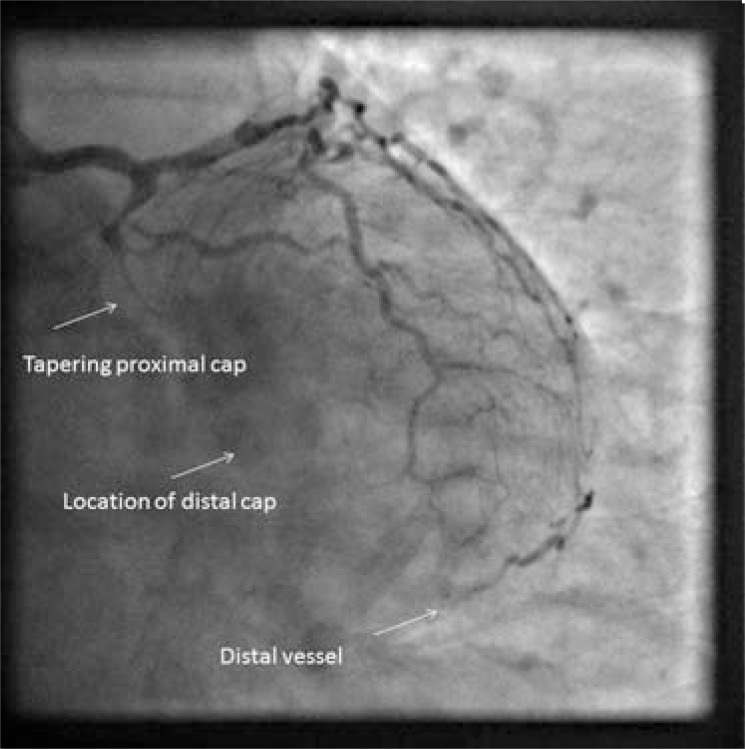
Still from PCI procedure, 1 year following angiogram in (Fig. 3). Long tapered segment replaced by blunt stump.

A tapered proximal end frequently leads the guidewire into a less resistant occlusion. Proximal tapering may be a consequence of ACS occlusion with limited retrograde thrombus propagation; perhaps a tapered stump is the point of occlusion from which thrombus has either not propagated, or has regressed to. It might be the angiographic appearance of histological non occlusive vessel. It may be an early appearance that tends to remodel towards the blunt stump appearance with time. (Figs. **3** and **4**).

**What determines penetrability of the occluded segment?**

**What determines extent of calcification of the occluded segment?**

**What determines recanalization / in segment neo vascularisation?**

These questions may relate to the extent of atheroma in the vessel in which the CTO has developed. One might speculate that an occlusion developing adjacent to multiple moderate to severe plaques will propagate, and subsequently remodel, and neovascularise differently to one which arises in a less diseased background.

## CHANGES WITH AGE

The timecourse of CTO morphology development is not known, as serial observations are difficult to achieve. Trends to increased calcification, identified by Srivasta *et al.*, [7] continued beyond twenty years, although 65% of CTOs in this study were younger than 5 years old. 

**Fig. (5):**
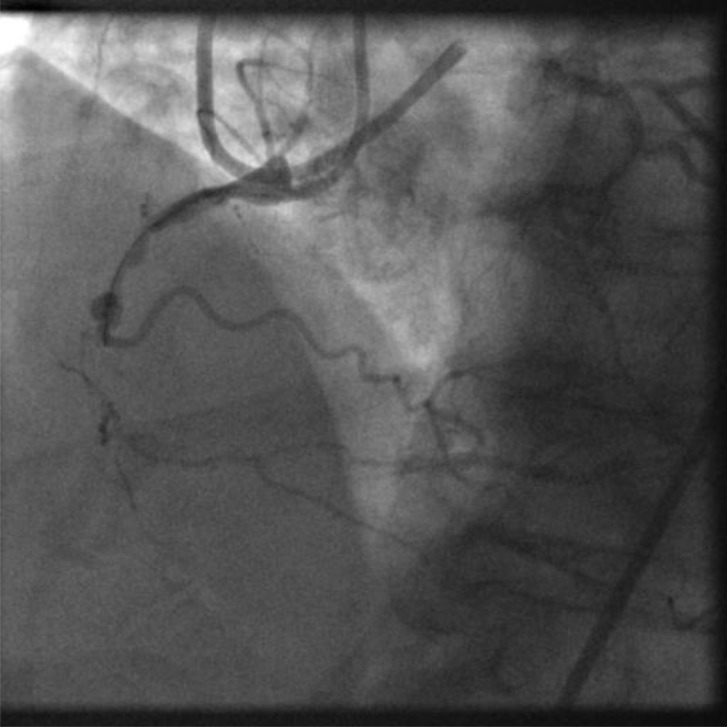
Still from coronary angiogram recorded during PCI in 2013 from patient in whom angina recurred 18 years post CABG. The occlusion of the RCA was reported on coronary angiography in 1993.

**Fig. (6):**
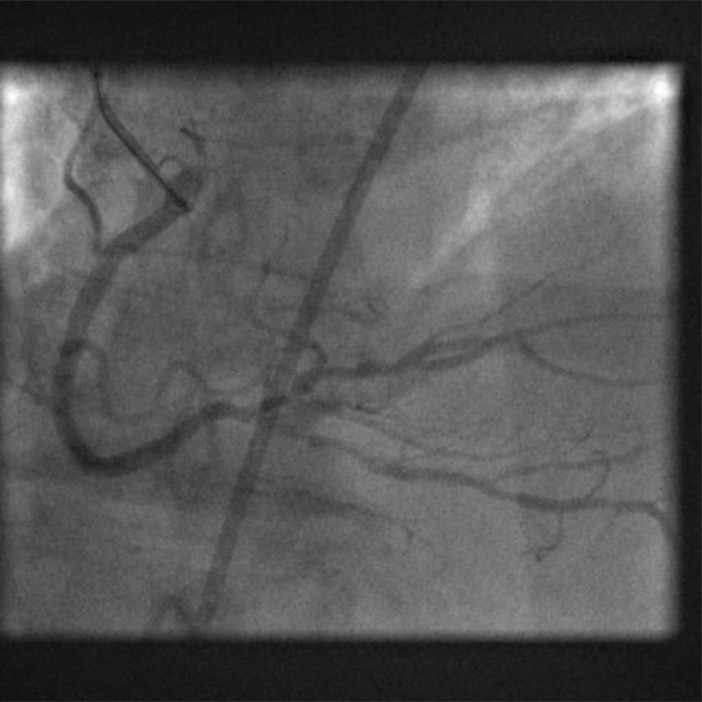
Coronary angiography of final result following successful PCI. 20 year old lesion crossed by antegrade wire escalation techniques.

Age of CTO occlusion is not now a strong indicator of a failed procedure [25]. Very old occlusions (greater than 20 years), [26], (Figs. **5** and **6**) can be highly amenable to PCI. We could speculate that the time course of active morphological changes occurs over a short number of years.

## SUMMARY OF MORPHOLOGY

At present we have greater practical experience of solving the problems presented by CTO morphology than knowledge of the developmental processes. More difficult CTO morphology with more resistant segments may well arise in previously diseased vessels, on the background of established atheroma. 

## COLLATERAL BIOLOGY

### Collateral Formation

It is widely accepted that collateral vessels originate as arterioles connecting the vascular beds of the visible coronary arteries [27]. In health these connections do not permit significant blood flow between territories. In the presence of coronary disease, significant pressure differences between variably diseased coronaries lead to dilatation of these vessels and thus flow. Increased endothelial shear stress mediates vessel remodelling through recruitment of smooth muscle cells, into mature arteries. 

### Studies of Influencing Factors

Physical exercise has a long association with benefit. Heberden described improved angina following a regime of wood chopping [28]. Human studies necessitate indirect evidence of collateral flow, but improvements in ECG parameters and thallium SPECT have been described following randomised trials of exercise training.

### Biological Mediators

There is a substantial literature on molecular mechanisms associated with collateral formation [27]. Preinfarction angina has been shown to predict collateral formation, assessed by emergency coronary angiography [29] Poor collateral networks have been demonstrated in patients with diabetes mellitus [30] and renal disease [31]. Genetic variations in VEGF sensitivity could be demonstrated in cultured myocytes from patients with or without angiographically detected collaterals [21]. Detailed phenotyping of IHD patients may well be a useful target for future genetic linkage studies. 

### How Protective are Collaterals?

Associations between greater availability of collaterals and decreased susceptibility to the consequences of IHD are well established [32], but not consistent [33]. A recent meta-analysis suggested a significant increase in survival with good collaterals [34] Occlusion without infarction is well described, but a spectrum exists. The OAT trial recruited patients with occluded vessels, akinetic wall motion and absence of inducible ischaemia, at least 72 hours after myocardial infarction. No benefit was seen in patients randomised to revascularisation after some months of follow up. The extent of collaterals did predict improved outcomes in this study [35], but did not influence the negative relationship in this study between intervention and outcomes.

A recent consecutive series of patients with CTO identified by coronary angiography in a single Korean institution were studied using Cardiac MRI. The analysis excluded patients with documented ACS within three months of the index angiogram to ensure that occluded vessels were CTOs, and patients with prior revascularisation to avoid detection of peri-procedural myocardial infarction. Evidence of prior myocardial infarction was detected in increasing frequency according to the sensitivity of the modality used. One quarter of patients had ECG Q waves, 42% of patients had a history consistent with prior myocardial infarction, MRI wall motion abnormalities were seen in 69%, late-gadolinium enhancement (LGE) was detected in 86% of patients. Interestingly the extent of collateral development predicted presence of Q waves, frequency of transmurality of LGE, and LGE volume [36]. However, many patients with evidence suggestive of significant infarction could have MRI findings compatible with potential benefit from revascularisation, approximately 20% patients with Q waves, and 30% of patients with poorly developed collaterals had less than 25% transmurality of LGE.

### What is the Maximum Extent of Collateral Flow?

Werner *et al*. has studied collateral function in a series of elegant investigations. Perhaps the most striking observation occurred in a series of 107 patients with occluded vessels of greater than one month duration [37]. This study analysed patients without ECG Q waves, nor history of prior MI. The protocol required the occlusion to be crossed with a wire then a microcatheter, to permit exchange for a pressure wire. FFR varied from 0.03 to 0.78, mean 0.32 ± 0.13.

These patients with occluded vessels without (detectable) infarction are likely to have readily developed collateral networks. Patients with infarction may have occluded the vessel faster than arteriogensis could compensate. The absence of sufficient collateral flow to prevent ischaemia in this population implies that ischemia is inevitable for viable segments downstream to an occlusion.

### Do Collaterals Predict Post PCI Outcomes? 

Two case series have shown no impact from collateral flow on degree of restenosis [38, 39].

### Collateral Regression Post PCI

Following successful PCI, diminishing collateral flow is generally observed during the procedure [37]. Reduction in flow is more rapid in intramyocardial vessels than large calibre epicardial collaterals. These channels do not regress completely, but protection against subsequent events in the previously occluded vessels, most notably stent thrombosis, is partial.

### Occluded Vessel Physiology

The goal of CTO PCI is restoration and maintenance of patency. Residual impairment of vessel function has been suggested by two recent studies. Galassi *et al*. investigated one hundred CTO cases by measuring quantitative coronary angiography (QCA) in the distal vessel following stent implantation, and again at follow up angiography approximately one year post PCI [40]. Sub groups of patients were further investigated using IVUS, or pacing to assess endothelial dependent vasomotor response. Distal vessel diameters increased measured by QCA, there was no change in vessel remodelling assessed by IVUS measurement of external elastic lamina dimensions distally. Immediately post procedure, neither pacing nor GTN caused detectable vasodilatation; at follow up pacing was associated with vasoconstriction and GTN with vasodilatation. The authors interpret their findings as demonstration of endothelial dysfunction that persists, although acknowledge that this is confounded by use of first generation drug eluting stents (DES), which have been shown to be associated with similar findings in non CTO vessels.

Brugaletta and co-workers investigated responses to intra-coronary administration of acetylcholine and GTN, representing endothelium dependent and independent mediators of vasodilation [41]. Nineteen patients were studied following successful stent implantation using a Doppler wire to assess flow and QCA to assess vessel diameters. Acetyl choline infusion was associated with vasoconstriction, and GTN vasodilatation, suggesting endothelial dysfunction. Intriguingly, less dysfunction was correlated with greater collateral circulation assessed pre procedure by collateral connection (CC) grade. These studies represent the first steps into a new field of investigation in which standardised methodologies, and greater statistical power should reveal valuable insights into occluded vessel biology.

### Pathophysiological Insights into the Hybrid Algorithm Approach to PCI

Consensus between highly experienced operators has established a structured approach to decision making in CTO PCI [42]. This algorithm integrates well studied variables that predict rapidly successful strategies. Procedure outcomes are excellent with high success rates and low complication rates [43].

It is assumed that a procedure will be performed following dual injections, and the tactics chosen will be honed by interpretation of high quality angiographic images. The pathophysiological features that underlie the angiographic characteristics of the decision making algorithm are discussed briefly.

### Lesion Length

Longer occluded segments are more difficult to cross than shorter; a threshold of 20mm was established by consensus in the Euro CTO Club [44] and by analysis in the J-CTO registry of model of CTO complexity [45]. This study looked at predictors of antegrade wiring within 30 minutes in 494 CTO lesions. Length greater than 20mm was a stronger predictor than calcification, tortuosity, blunt stump, or previous failure.

### Cap Ambiguity 

Angiographic ambiguity at the proximal edge reflects the combined challenge of the resistant proximal cap, and geographic uncertainty generally resulting from multiple side branches. The vessel track may plausibly follow a broad angle of tracks. A resistant cap in a straight segment may be overcome (or outflanked) by a penetrative wire. Manipulation of such a wire in an ambiguous architecture would represent a high risk of failure and perhaps perforation.

### Target Vessel Beyond the Distal Cap

Small and irregular vessels downstream of occluded segments may represent underfilling, profound spasm, or diffuse atheroma. We have seen that it has been difficult to identify the distal cap by histology or IVUS, and that determinants of morphology are not known. In any case, a small irregular vessel is difficult to enter whether by antegrade techniques, either wiring or dissection-re-entry, and is a reason to consider the retrograde approach.

### Interventional Collateral

Biological determinants of interventional utility have not been studied in depth. A large volume US series reported 68% successful septal crossing, 24% epicardial and 8% via a bypass graft. Failure to cross a collateral is predicted by tortuosity [46]. This topic is considered in greater depth in a companion article in the series.

## CONCLUSIONS

The studies of pathophysiology of CTO are small, and clinical experience of CTO PCI outnumbers patients studied by post mortem or by IVUS. This narrow knowledge base has however enabled marked progress in CTO PCI, transforming procedural outcomes. Experienced operators use the hybrid algorithm to enable a structured approach to approach every lesion expecting success. Wires can successfully traverse weaker channels within the occluded lumen, or the potential space beneath the intima can be exploited by use of knuckled wires in combination with re-entry technology. Further technical developments are likely to be aimed at shortening procedure times.

Future studies of CTO morphology may yet yield novel insights into the processes of lesion development. For example analysis of CTO morphology in patients for whom coronary angiography predating CTO formation is available, might allow a deeper interpretation of IVUS data with regard to position and constitution of the elusive distal cap. Expanding the knowledge base may well lead to further disruptive technologies in this fascinating arena.

## ABBREVIATIONS

ACS: = Acute Coronary Syndrome
CTO: = Chronic total occlusion
DES: = Drug eluting stent
GTN: = Glycerol Tri-Nitrate
IVUS: = Intra vascular ultrasound
IVUS-VH: = Virtual histology IVUS
J-CTO: = Multicenter CTO registry in Japan
LAD: = Left Anterior Descending Coronary Artery
LGE: = Late-gadolinium enhancement
OAT: = Occluded Artery Trial
PCI: = Percutaneous Cornoary Intervention
QCA: = Quantitative Coronary Angiography
RCA: = Right Coronary Artery
STRAW: = Subintimal Transcatheter Withdrawal
